# Sexual abuse and psychotic phenomena: a directed acyclic graph analysis of affective symptoms using English national psychiatric survey data

**DOI:** 10.1017/S003329172300185X

**Published:** 2023-12

**Authors:** Giusi Moffa, Jack Kuipers, Elizabeth Kuipers, Sally McManus, Paul Bebbington

**Affiliations:** 1 University of Basel, Basel, Switzerland; 2 Department of Biosystems Science and Engineering, Eidgenossische Technische Hochschule Zurich, Basel, Switzerland; 3 King's College London, London, UK; 4 City University of London, London, UK; 5 University College London, London, UK

**Keywords:** Affective symptoms, DAGs, psychosis, sexual abuse

## Abstract

**Background:**

Sexual abuse and bullying are associated with poor mental health in adulthood. We previously established a clear relationship between bullying and symptoms of psychosis. Similarly, we would expect sexual abuse to be linked to the emergence of psychotic symptoms, through effects on negative affect.

**Method:**

We analysed English data from the Adult Psychiatric Morbidity Surveys, carried out in 2007 (*N* = 5954) and 2014 (*N* = 5946), based on representative national samples living in private households. We used probabilistic graphical models represented by directed acyclic graphs (DAGs). We obtained measures of persecutory ideation and auditory hallucinosis from the Psychosis Screening Questionnaire, and identified affective symptoms using the Clinical Interview Schedule. We included cannabis consumption and sex as they may determine the relationship between symptoms. We constrained incoming edges to sexual abuse and bullying to respect temporality.

**Results:**

In the DAG analyses, contrary to our expectations, paranoia appeared early in the cascade of relationships, close to the abuse variables, and generally lying upstream of affective symptoms. Paranoia was consistently directly antecedent to hallucinations, but also indirectly so, via non-psychotic symptoms. Hallucinosis was also the endpoint of pathways involving non-psychotic symptoms.

**Conclusions:**

Via worry, sexual abuse and bullying appear to drive a range of affective symptoms, and in some people, these may encourage the emergence of hallucinations. The link between adverse experiences and paranoia is much more direct. These findings have implications for managing distressing outcomes. In particular, worry may be a salient target for intervention in psychosis.

## Introduction

This paper links the psychiatric impact of sexual abuse with recent network models of psychosis, and it may have implications for psychological therapies for psychosis targeting ancillary affective symptoms.

Current accounts of the origins of psychosis acknowledge contributions from biological, psychological and social domains (Broome et al., 2005; Howes & Murray, 2014; Zwicker, Denovan-Wright, & Uher, 2018). Experiences that involve abuse, threats, intrusiveness, disparagement, and an insistence on acting in ways distressing to the recipient appear to have a particularly malign impact on the emergence of psychotic symptoms (Arseneault et al., 2011; Bentall, Wickham, Shevlin, & Varese, 2012; de Vries et al., 2019; Kelleher et al., 2013; Varese, Barkus, & Bentall, 2012). Salient examples include bullying and sexual abuse, which almost invariably display all of the elements above, and are hence likely to have significant psychological and psychiatric consequences. These may also be mediated by the particularly damaging effects of childhood trauma on social cognition, which may form a pathway leading to major adult psychiatric disorders (Dauvermann et al., 2021; Rokita, Dauvermann, & Donohoe, 2018; Rokita et al., 2020).

Sexual abuse is evidently common. British-based findings of 11% (female) and 5% (male) for contact sexual abuse (Bebbington et al., 2011) are closely similar to those found elsewhere (Coxell, King, Mezey, & Gordon, 1999; May-Chahal & Cawson, 2005). In addition, early sexual victimisation increases vulnerability to further abuse, thereby compounding its impact (Classen, Palesh, & Aggarwal, 2005; Cloitre, Tardiff, Marzuk, Leon, & Portera, 1996; Coxell et al., 1999; Muenzenmaier, Meyer, Struening, & Ferber, 1993). In one study, childhood non-consensual sexual intercourse was associated with a ninefold increase in the odds of non-consensual sexual abuse in adulthood (Bebbington et al., 2011). Thus people reporting sexual abuse will often have been subjected to repetitive and continuing harmful acts.

Sexual abuse appears to increase the likelihood of both non-psychotic and psychotic disorders (Jonas et al., 2011), and it is possible that these outcomes emerge through a common process. There is a clear association between non-psychotic symptoms (anxiety, worry, depression, mood instability, insomnia) and psychotic phenomena (paranoia, hallucinations) at a symptom level (e.g. Freeman et al., 2012; Sheaves et al., 2016). There is also evidence to indicate that affective symptoms may constitute the point of entry for the link between sexual abuse and psychosis (Bebbington et al., 2011; Marwaha & Bebbington, 2015). While the involvement of affective symptoms in mechanisms leading to schizophrenia has long been argued (e.g. McReynolds, 1960), interest has been renewed by the recent development of psychological treatments of psychosis (Craig et al., 2018; Freeman et al., 2015, 2017; Garety et al., 2021), several of which have beneficial effects on affective phenomena.

Direct intervention studies remain the gold standard for causal inference in putative causal systems, but are expensive and not always feasible. It is therefore sensible to prioritise proposed interventions in relation to their likely effectiveness. Attempts have consequently been made to optimise methods of causal inference in datasets that do not involve interventions. Psychological studies have traditionally employed varieties of logistic regression to study mediation in cross-sectional datasets (e.g. Catone et al., 2015; Marwaha, Broome, Bebbington, Kuipers, & Freeman, 2014). While these give a provisional idea of the plausibility of candidate mediators, they do so by identifying variables that may not in fact meet the criteria for mediation. Progress in statistical methods enables a more complete characterisation of complex relationships, by jointly describing the connection between a larger number of variables. Network analysis, including association networks, partial correlation networks, and relative importance networks, seeks to describe psychopathology as the interplay of different symptoms (McNally, 2016). Applied to mental disorders, they treat the symptom picture as a system of individual interacting variables, and have indeed sometimes been taken as removing any need for invoking an underlying disorder (Borsboom & Cramer, 2013; Fried et al., 2017; Isvoranu, Borsboom, van Os, & Guloksuz, 2016; Isvoranu et al., 2017; Klippel et al., 2018; McNally, 2016; McNally et al., 2015; Murphy, McBride, Fried, & Shevlin, 2018; Piao et al., 2021; van Rooijen et al., 2018; Wigman, de Vos, Wichers, van Os, & Bartels-Velthuis, 2017). This has encouraged the idea that non-psychotic symptoms may shape the relationship between adversity and psychotic experiences as moderators and mediators. Such symptoms might then serve as candidate targets for psychological treatments.

However, while these approaches all describe the strength and sign of association and may produce suggestive results, they are not capable of determining causality. We have consequently argued for the superiority of probabilistic graphical models based on directed acyclic graphs (DAGs) for performing causal analyses in psychological research (Kuipers, Moffa, Kuipers, Freeman, & Bebbington, 2019; Moffa et al., 2017, 2021). These are a form of Bayesian network able to model the overall dependence structure of multiple variables. Kuipers and Moffa (2017) have developed, and later modified to improve efficiency (Kuipers, Suter, & Moffa, 2022), a novel Bayesian method for sampling graphical structures from a posterior distribution. By implementing the do-calculus (Pearl, 2009) on each DAG from a sampled ensemble we can then predict the distribution of causal effects of a variable on another. We have previously used this strategy to investigate mechanisms linking bullying with persecutory ideation and hallucinations, based on data from the 2000 and 2007 British National Surveys of Psychiatric Morbidity (Moffa et al., 2017). In the current paper, we present an equivalent analysis of the impact of sexual abuse. We predict that sexual abuse, whether in childhood or in adult life, engenders psychotic symptoms specifically through its effect in eliciting affective symptoms. We conducted separate analyses on the two latest English Adult Psychiatric Morbidity Surveys carried out in 2007 and 2014 (McManus, Bebbington, Jenkins, & Brugha, 2016; McManus, Meltzer, Brugha, Bebbington, & Jenkins, 2009), chosen because they used the same detailed methods for establishing the experience and timing of sexual abuse (Bebbington, 2018). Given our earlier analysis of the effect of bullying (Moffa et al., 2017) and the overlap between the experience of bullying and sexual abuse, we have also included bullying in the current analyses. Furthermore, we included sex, as it may play an important role in determining the way symptoms interact with each other.

## Survey methods

### Setting, design, and participants

The third and fourth Adult Psychiatric Morbidity Surveys were carried out in 2007 and 2014. In each, participants were selected to be representative of those members of the population of England aged 16 and over, and living in private households. The sampling was constructed around postcode sectors chosen to be representative, between them, of the whole English population. Addresses were then selected at random from these postcode sectors. A detailed description of this procedure is available in the individual reports (McManus et al., 2016, 2009) and the Data Resource Profile (McManus et al., 2020). The overall response rate in both surveys was 57%. In 2014 respondents over 70 were not asked an item used in the analysis from the SCID-II BPD screen on mood instability, so in the interests of consistent comparison, we truncated both datasets to include only those aged 16 to 69 years. This reduced the numbers participating to 5954 in the 2007 survey, and 5946 in 2014.

### Measures

The assessment of persecutory ideation was based on question PSQ3a in the Psychosis Screening Questionnaire (PSQ; Bebbington & Nayani, 1995): ‘Have there been times when you felt that people were deliberately acting to harm you or your interests?’ Auditory hallucinations were identified from question PSQ5a: ‘Have you at any time heard voices saying quite a few words or sentences when there was no-one around that might account for it?’ These items were assessed in relation to the past year.

The Clinical Interview Schedule Revised (Lewis, Pelosi, Araya, & Dunn, 1992) was used to identify thirteen symptoms characteristic of non-psychotic psychiatric disorder lasting at least two weeks in the preceding month. Severity was then established in relation to the week before assessment. In addition, the trait of mood instability was identified from an item adopted from the DSM-IV Borderline Personality Disorder section of the Structured Clinical Interview for DSM-IV (SCID-II; First, Gibbon, Spitzer, Williams, & Benjamin, 1997; Marwaha et al., 2014): respondents were asked if they had experienced a lot of sudden mood changes in ‘the past several years’. Given the consistent implication of cannabis use in the development of psychotic disorders (Murray et al., 2017), we also included lifetime and past year use of cannabis.

In both surveys, a history of sexual abuse was obtained through a Computer-Assisted Self-completion Interview (CASI). A laptop computer was passed to the respondent, and the interviewer explained the procedure, making it clear that they would be unable to see the results of the self-completed parts of the interview. Information was sought about different degrees of sexual abuse: uncomfortable sexual talk; sexual touching; and full sexual intercourse. We defined abuse here as experiences of sexual touching or sexual intercourse that were non-consensual. Further questions dated the abuse, in particular its first occurrence. It was thus possible to distinguish sexual abuse occurring in childhood and adolescence (i.e. before 16 years) from that occurring in adulthood (i.e. 16 and over). Bullying was captured using an item from the List of Threatening Experiences measure which asked if respondents had ever experienced any of these problems or events at any time in their lives (Brugha, Bebbington, Tennant, & Hurry, 1985).

All variables were analysed in binary form (present v. otherwise), as required for the DAG procedures employed.

### Statistical procedure

The core assumption in using DAGs as causal diagrams is that the modelled variables have the capacity to represent the underlying causal mechanism. More specifically, the validity of causal diagrams relies on a property known as the Causal Markov condition which requires that the variables on the graph satisfy the probability independence relationships the DAG implies. Fundamental for network learning from data is also that *faithfulness* holds, meaning that a causal DAG captures all and only the conditional independence relationships that hold in the probability distribution of the modelled variables. Structure learning further requires that there are no unknown common causes of any two variables in the data (common cause confounding), nor any variables that determine selection into the dataset (selection bias). *Nodes* (the specific variables being analysed) are linked by *edges*, lines representing the directions of effect. These conventions can be seen in the figures summarising our results. A direct edge in the graph indicates a direct cause. Likewise, the presence of a *directed pathway* from one variable to another is also indicative of a causal link, albeit one with an indirect effect acting through intermediary variables on the path. The *parents* of a node are those immediately above it in the causal chain, while the *children* of a node are those immediately below.

For the DAG discovery phase of the current analysis, we employed a sampling algorithm consisting of a hybrid version of the partition Markov Chain Monte Carlo (MCMC) method (Kuipers & Moffa, 2017; Kuipers et al., 2022). All algorithms are available in the open-source R package BiDAG (https://CRAN.R-project.org/package=BiDAG; Suter, Kuipers, Moffa, & Beerenwinkel, 2023). We opt for this algorithmic approach and package, since this is a well-regarded implementation for fully Bayesian graphical structure learning (Kuipers et al., 2022; Suter et al., 2023). Additionally it benefits from demonstrated favourable performance for relatively large graphs (Rios, Moffa, & Kuipers, 2021).

To predict the putative range of causal effects we also followed a Bayesian strategy, as implemented by (Moffa et al., 2017) for binary variables, and now also available in the Bestie R package (https://CRAN.R-project.org/package=Bestie). The estimation proceeds by drawing a sample of DAGs, and evaluating for each of them how manipulating one variable affects another. Two notable features characterise the method. First, it captures and describes both the *strength* and the *direction* of causal effects. Secondly, it characterises the inference variability stemming from both the parameter and the structural uncertainty, whereby several different graphical arrangements of variables may each be capable of explaining the data reasonably well. Sampling from the range of possible DAGs in proportion to their posterior distribution before determining the compatible causal effects from each of them captures the ensuing uncertainty. For each analysis, we sampled a total of 10 000 DAGs. For fuller details of the method in action we refer the reader to Kuipers et al., 2019; Moffa et al., 2017.

In the DAGs presented in this paper we applied some constraints to reflect domain knowledge about the phenomenon under study: childhood sexual abuse and bullying, referred to events that were temporally antecedent to the assessment of the psychological variables, hence they only admit incoming edges from sex and from each other. For the same reason, adult sexual abuse only admits incoming edges from sex, bullying and earlier abuse, while sex only admits outgoing edges since symptoms cannot cause it.

Some pairs of variables display very similar association patterns (online Supplementary Figures 1 and 2), a feature which complicates learning DAG structures. To overcome the difficulty we combined the pairs of variables which consistently displayed the highest level of similarity in their correlation pattern for both survey instances (2007, online Supplementary Figure 1 and 2014, online Supplementary Figure 2). The top three pairs are stable in coming closer together in both datasets, therefore we created three composite variables from them which took the value 1 whenever either component was 1 (Depression from either Depressive ideation or Depression; Cannabis from either Cannabis ever or in the past year; Worry from either Worry or Anxiety). Finally, we also included sex as an upstream node to account for any ensuing differences in the underlying mechanism.

## Results

We found that the reported prevalence of sexual abuse was remarkably similar in the 2007 and 2014 surveys, being more than twice as frequent in females. Of female respondents, 11.3% reported sexual abuse *before* the age of 16 in 2007, and 10.3% in 2014. The equivalent figures in males were 5.3% and 4.3%. Sexual abuse *after* the age of 16 was reported by 8.6% of females in 2007 and by 8.5% in 2014, compared with figures of 1.9% and 2.6% in males. The early experience of sexual abuse was associated with further sexual abuse in adulthood: in both surveys, a quarter of people abused in childhood also reported later abuse. Likewise, a history of bullying in childhood was linked to adult sexual abuse.

**Figure. 1.**
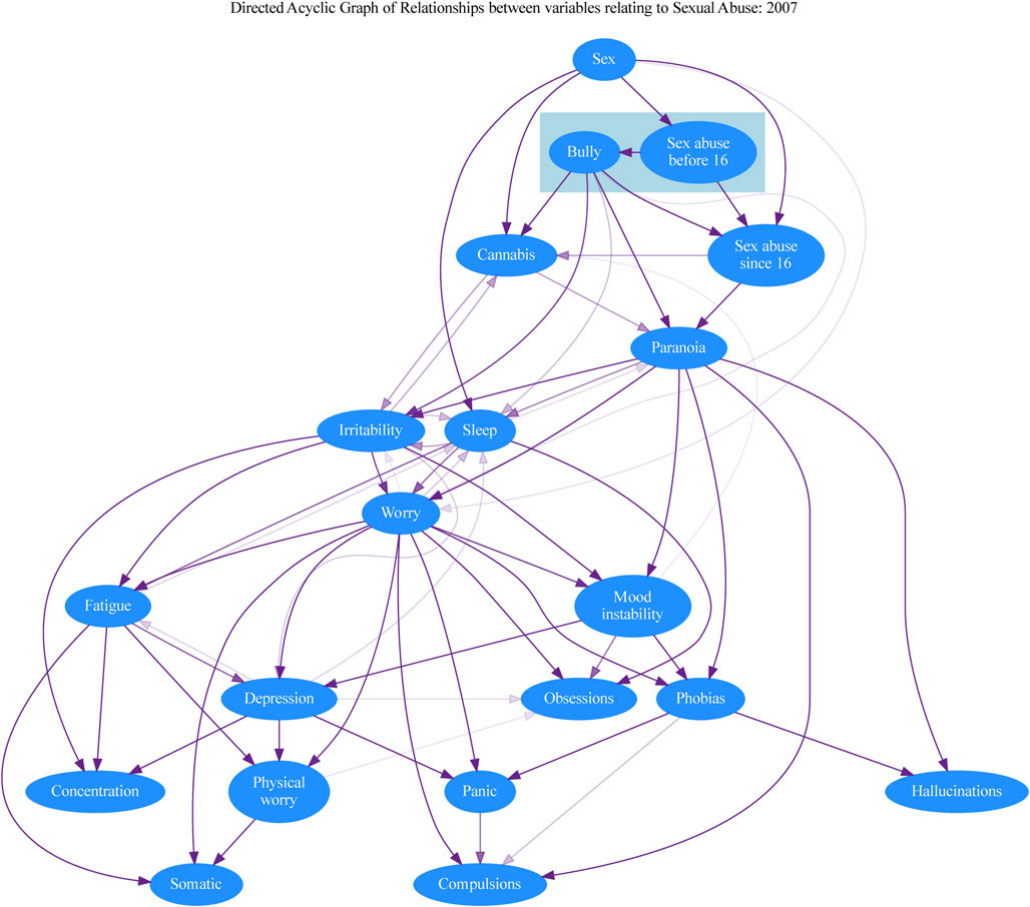
Consensus graph summarising the posterior distribution of DAGs for the 2007 survey data. The colour intensity of each edge is proportional to the frequency with which each edge appears in a sample of 10 000 DAGs. For clarity and to highlight the stronger relationships, the figure only displays edges appearing in at least 10% of the sampled DAGs.

Figures 1 and 3 show the consensus graphs generated by the DAG sampling procedure. To highlight the stronger relationships, consensus graphs only display edges that appear in at least 10% of the sampled DAGs. The colour intensity of each edge is proportional to the frequency with which it was present among the sampled DAGs, a way of visualising the degree of support for the presence of each edge with the given direction. The presence of edges going in opposite directions is indicative of situations where the data did not uniquely identify one direction. As indicated above, the presence or absence of sexual abuse is set as an antecedent in both analyses, with childhood sexual abuse antecedent to adult sexual abuse in the DAG.

**Figure. 2.**
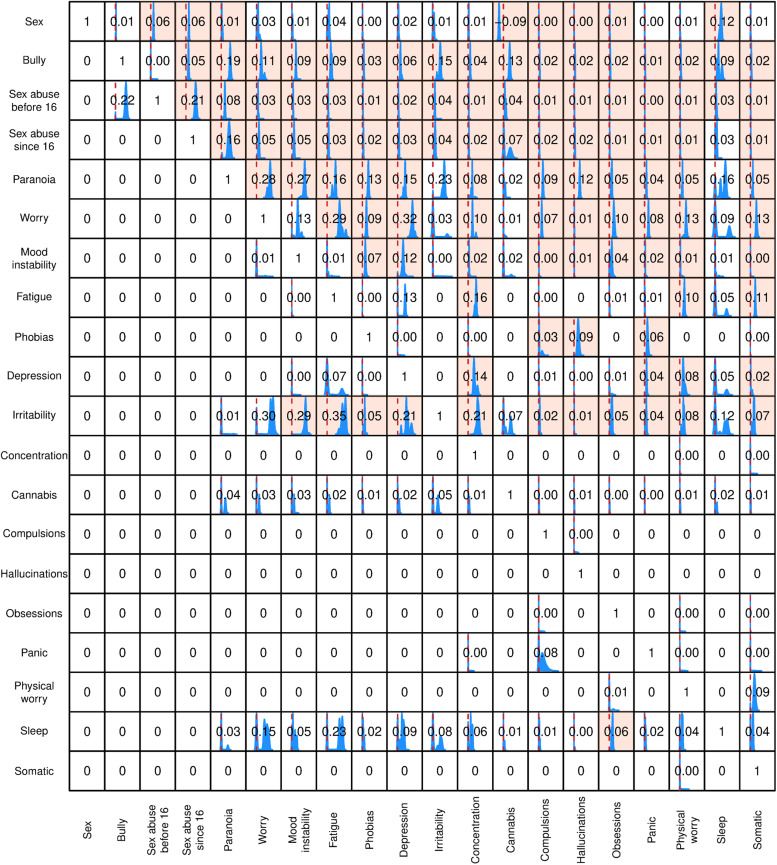
Posterior distribution of causal effects, on the scale of risk differences, compatible with the survey data from 2007. For each sampled DAG the procedure evaluates intervention effects and the figure displays histograms of the values from all DAGs (where the x-axis for all boxes ranges from −0.2 to 0.5). The red vertical line indicates zero risk difference. The numbers in the box express the average causal effect, with zero indicating no effect, and the box shading highlights cases where the 95% credible interval does not include zero.

In Figs 2 and 4, we show the posterior distributions of causal effects, on the scale of risk differences, between the variables for the 2007 and 2014 surveys respectively, as derived from the full ensemble of sampled DAGs and compatible with the observed data. The red vertical line in each box indicates zero causal effect. In the cases where the 95% credible interval (the Bayesian counterpart of confidence limits) does not cover the point corresponding to zero causal effect we shaded the whole box. The numbers in the boxes indicate the relevant *average* causal effect (or risk difference), with a zero where there was no effect between the two variables.

**Figure. 3.**
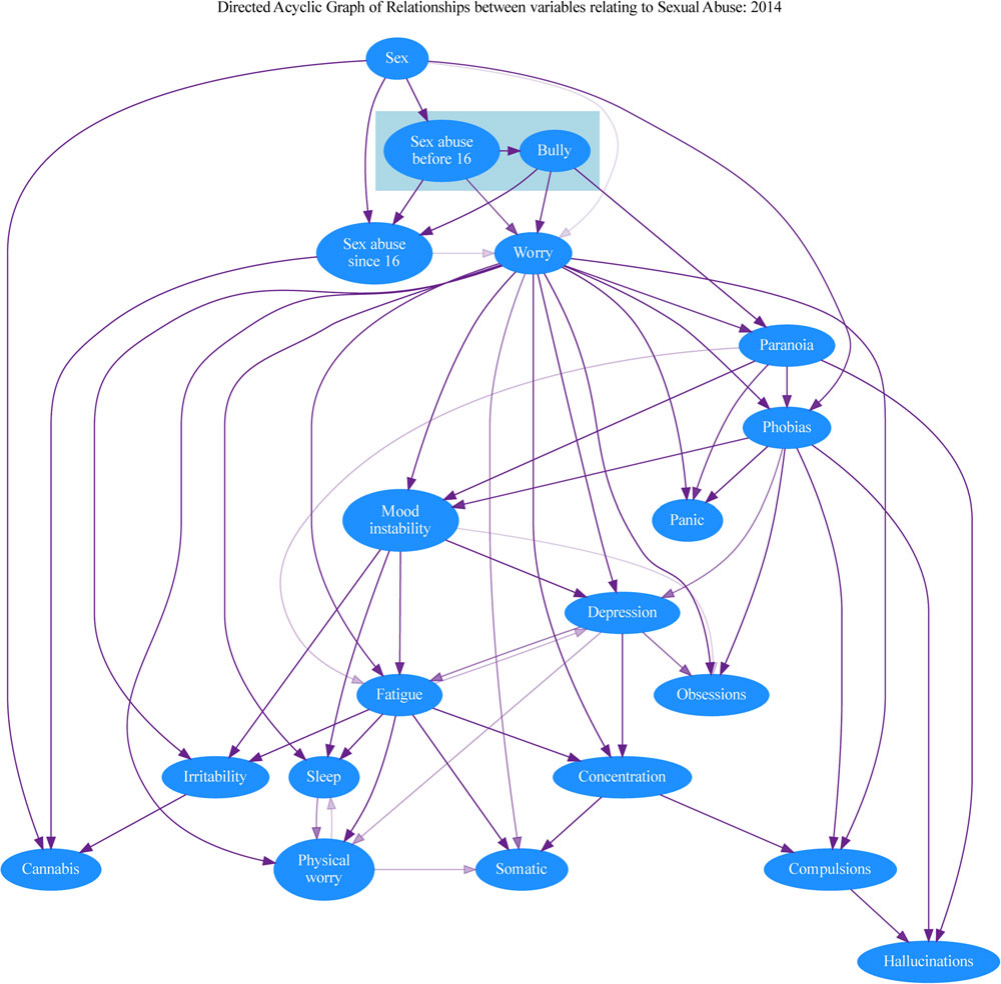
Consensus graph summarising the posterior distribution of DAGs for the 2014 survey data. The colour intensity of each edge is proportional to the frequency with which each edge appears in a sample of 10 000 DAGs. For clarity and to highlight the stronger relationships, the figure only displays edges appearing in at least 10% of the sampled DAGs.

Even in datasets of this size there is still a possibility that inconsistency will arise due to random variability in the detailed relationships between large numbers of variables. This is of course a strong argument for our use of more than one survey. Nevertheless, although there are differences between the DAGs displayed in Figs 1 and 3, there are also consistent patterns with interesting implications.

**Figure. 4.**
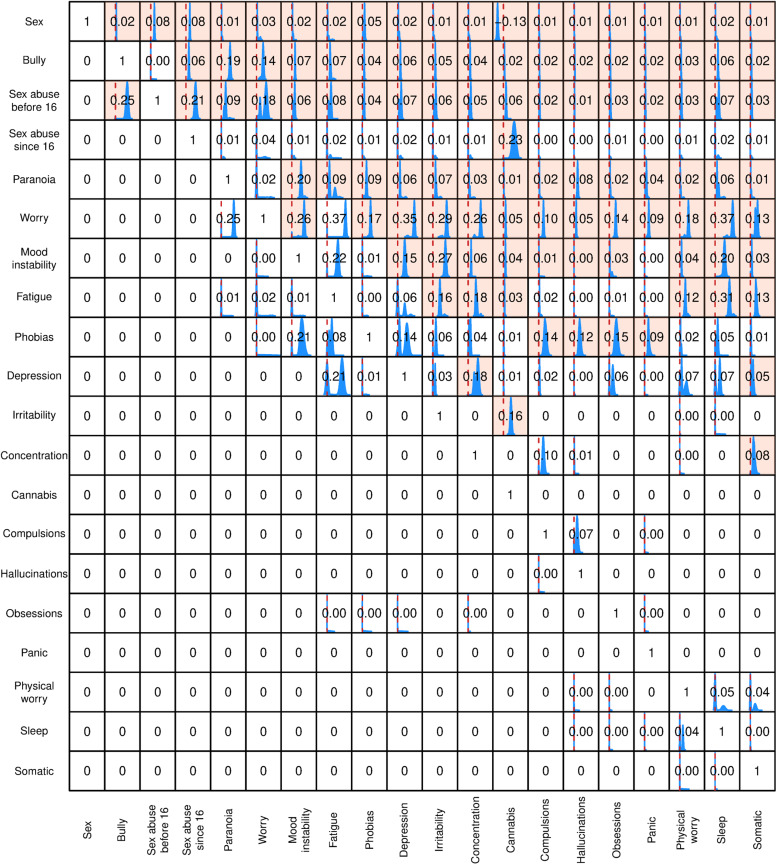
Posterior distribution of causal effects, on the scale of risk differences, compatible with the survey data from 2014. For each sampled DAG the procedure evaluates intervention effects and the figure displays histograms of the values from all DAGs (where the x-axis for all boxes ranges from −0.2 to 0.5). The red vertical line indicates zero risk difference. The numbers in the box express the average causal effect, with zero indicating no effect, and the box shading highlights cases where the 95% credible interval does not include zero.

The greater frequency of sexual abuse in females is reflected in both DAGs, and early sexual abuse clearly increases the likelihood of both further abuse and bullying. The propensity to paranoia brought about by early sexual abuse may be increased by further sexual abuse after the age of 16, though this effect was only apparent in the 2007 dataset. A causal link between the two types of abuse and paranoia is apparent in both datasets, although in the 2014 data this is a weak effect and partly mediated by worry.

Interestingly, and counter to our expectations, paranoia is placed *early* in the cascade of relationships, close to the abuse variables. Consequently, in both datasets, paranoia lies upstream of a range of affective symptoms. In addition to indirect links mediated by these various affective pathways (notably via phobic symptoms), paranoia was consistently a *direct* antecedent of hallucinations. Hallucinations also seem to be the endpoint of pathways involving several nonpsychotic symptoms, while the relationship between paranoia and hallucinations was strengthened by the presence of phobias and compulsions. The analyses thus suggest that sexual abuse and bullying have important relationships with the emergence of a range of affective symptoms, such as worry and mood instability, and that in some people these in turn encourage the emergence of hallucinations. In contrast, the link between these adverse experiences and paranoia is more direct. In both datasets, worry (a symptom that combines within itself affective and cognitive elements) seems to encourage the emergence of many of the non-psychotic symptoms and precedes paranoia in the 2014 data set. However, in the 2007 data set paranoia appeared as the parent to worry.

The implications of our analysis for the significance of cannabis use in psychosis are of particular interest when we set sexual abuse and bullying as antecedent effects. In both sets, cannabis use is downstream of bullying and sexual abuse, implying its use is more common in people who have had those experiences. This was partly mediated by worry in 2014, but not in 2007. The position of cannabis use is of particular interest, given the widely accepted view that it has a separate and causal role in the generation of psychotic disorders (Murray et al., 2017). In the 2007 dataset it is downstream only of gender and a history of abuse but has no links with any psychiatric symptoms. Cannabis use is again linked to the abuse items in the 2014 dataset, but from there it only has weak direct links to irritability and paranoia.

## Discussion

We applied DAG analysis to investigate the relationship between sexual abuse and bullying with affective and psychotic symptoms. We incorporated the effect of bullying in the current analyses alongside our measures of sexual abuse because of its important effects highlighted by our DAG analysis of data from the 2000 and 2007 National Surveys of Psychiatric Morbidity to examine the links between bullying and psychosis (Moffa et al., 2017).

DAGs are a particular form of network analysis, and we have argued elsewhere for their advantages in epidemiological psychiatry (Kuipers et al., 2019; Moffa et al., 2017, 2021). Under specific conditions, they are capable of determining the relative likelihood and strength of causal relationships. There are restrictions: causal inference is only fully justified if the joint distribution of the variables satisfies *all* the conditional independence relationships encoded by the DAG and *only* those relationships. Moreover, results may be affected by unmeasured confounders. Nevertheless, when the focus is on a causal perspective, DAGs represent a considerable advance over other recent applications of network approaches to complex interactions based on partial correlations between variables (Fried et al., 2017; Isvoranu et al., 2016, 2017; McNally et al., 2015; Wigman et al., 2017): in these approaches, the *absence* of an edge would refute a direct causal link between two variables, but the *existence* of edges cannot provide information on the direction of effects. Furthermore, causal mechanisms exist which a DAG can represent but an undirected graphical model cannot; one important example is the so called v-structure where two variables cause a third, but with no direct link between them (e.g. A → B ← C). A detailed discussion of the use of undirected models in exploratory analyses of psychological datasets appears in Epskamp, Waldorp, Mõttus, and Borsboom, 2018.

Theoretical models have linked a range of affective variables to paranoia, with the suggestion that affective processes encourage the emergence of psychotic symptoms (Freeman, 2016). This was the origin of our hypothesis that affective variables would be antecedent to both persecutory ideation and hallucinations, also in line with the associations reported by (Bentall et al., 2012). However, the findings presented here suggest that paranoia may be the *direct* consequence of early abuse involving bullying or sexual exploitation. This is plausible, as it implies that the abuse shapes cognition, and that this then leads to affective consequences. There is some support for effects of abuse on social cognition in psychosis (Dauvermann et al., 2021). In contrast, hallucinosis appears to be a downstream consequence of paranoia, but is also downstream of a wide range of affective symptoms.

Worry seems to be central to the development of other affective symptoms. Obsessions, compulsions, somatic symptoms and worry focussing specifically on physical symptoms are consistently downstream. Panic seems particularly related to worry and phobic symptoms. There are nevertheless discrepancies between the two datasets in the detailed relationships between individual affective symptoms. This potentially highlights the need to study the role of worry in more detail, for which longitudinal measurements might be especially relevant.

Sleep disturbance is common in people with psychotic symptoms and it has been argued that disrupted sleep encourages the emergence of psychotic experiences, rather than merely being a secondary consequence. In our data however poor sleep appears to be the direct or indirect consequence of paranoia. It should be noted nevertheless that sleep hygiene interventions have reported improvement in psychotic symptoms (Freeman et al., 2017; Reeve, Emsley, Sheaves, & Freeman, 2018).

The findings relating to cannabis use in the year before the assessment were counter to our expectations and at variance with the accepted view in the literature (Murray et al., 2017). Cannabis use was linked to abusive experience in both datasets, but it had only the weakest downstream links to any psychological variables in 2007 and none at all in the 2014 dataset.

It is difficult to argue that the inconsistencies in results between the two data sets are due to different methodologies or the representativeness of the samples. The central purpose of the English Adult Psychiatric Morbidity Survey programme is to record secular changes in the prevalence of psychiatric disorders and to relate these, at least tentatively, to social developments. For this reason, the two national surveys forming the basis of the current paper used identical sampling procedures and virtually identical methods of enquiry, thus enabling an instant test of reproducibility. The samples are large, and the sampling methods and weighting procedures render the data closely representative of the general population of England living in private households. There was in fact a secular increase in the overall prevalence of psychotic disorder between the surveys: this increase, from 0.4% to 0.7%, which is quite large, may have been sufficient to add complexity to the 2014 DAG analysis and account for some of the inconsistencies. We have previously shown that these data sets can provide consistent results from DAG analyses (Moffa et al., 2017), so it is unclear whether the differences found here were due to changes in observed prevalence, unmeasured confounders, or both. For example, societal and political changes over time may impact on the observed relationships.

Another methodological issue that should be considered is the question of whether the rate of reportage of sexual abuse is dependent on individuals’ position on a spectrum of mental well-being: this would encourage a spurious inference of causal direction. However, this is at odds with the published finding from the 2007 survey that reports of *re-victimisation* were no more likely in people identified as having a mental disorder (Bebbington et al., 2011). Moreover, the effectiveness of the CASI technique for eliciting detailed and sensitive sexual information is supported by the very consistent prevalence of sexual abuse in the two surveys, despite an intervening period in which the general public became considerably more aware of the fact, frequency and consequences of such abuse.

## Conclusions

We have previously established the utility of DAG analyses in improving our understanding of the relationships between complex variables in psychotic disorders and for informing targeted interventions to improve outcomes for people with these problems. We now demonstrate this in the broader context of sexual abuse and affective symptoms. In particular, sexual abuse and bullying appeared to be closely linked to both paranoia and hallucinations, and the pathways, although not entirely consistent between the 2007 and 2014 data sets, confirm the importance of considering a wide range of potential sequelae of such adverse environmental precursors. Worry may be a particularly salient target for intervention.

## Supporting information

Moffa et al. supplementary material Moffa et al. supplementary material

## Author and article information

From the Division of Psychiatry, University College London UK; Department of Mathematics and Computer Science, University of Basel, Basel, Switzerland; ETH Zurich, Basel, Switzerland; NatCen Social Research, London UK; City, University of London, London UK; King's College London, Department of Psychology, Institute of Psychiatry, Psychology and Neuroscience, London UK; NIHR Biomedical Research Centre, South London and Maudsley NHS Foundation Trust, Beckenham, UK.

## Supplementary material

The supplementary material for this article can be found at https://doi.org/10.1017/S003329172300185X.
