# Sexual abuse and psychotic phenomena: a directed acyclic graph analysis of affective symptoms using English national psychiatric survey data – ERRATUM

**DOI:** 10.1017/S0033291723002398

**Published:** 2023-12

**Authors:** Giusi Moffa, Jack Kuipers, Elizabeth Kuipers, Sally McManus, Paul Bebbington

The Psychological Medicine article ‘Sexual abuse and psychotic phenomena: a directed acyclic graph analysis of affective symptoms using English national psychiatric survey data’ was missing an orcid for one of the authors and had incorrect affiliation information. The publisher apologises for these errors.
