# Supplementary material for: Sexual abuse and psychotic phenomena: a directed acyclic graph analysis of affective symptoms using English national psychiatric survey data
Source: Psychol Med. 2023 Jul 24;53(16):7817–26. doi: 10.1017/S003329172300185X (PMC10755243; doi:10.1017/S003329172300185X)
Supplement: Moffa et al. supplementary material [file S003329172300185Xsup001.pdf]

Supplementary Material

Pairs of variables with highly similar correlation patterns may lead to difficulties in separating their corresponding nodes on the graphical diagram. Therefore for the DAG analysis we combine each of the three pairs with the most similar patterns, based on the Euclidean distance between correlation vectors, into a single compound variable. In our analysis, this is the case for the following pairs: CanEver (Cannabis ever) and CanYear (Cannabis in the past year), Anxiety and Worry, DepIdeas (Depressive ideation) and Depression.

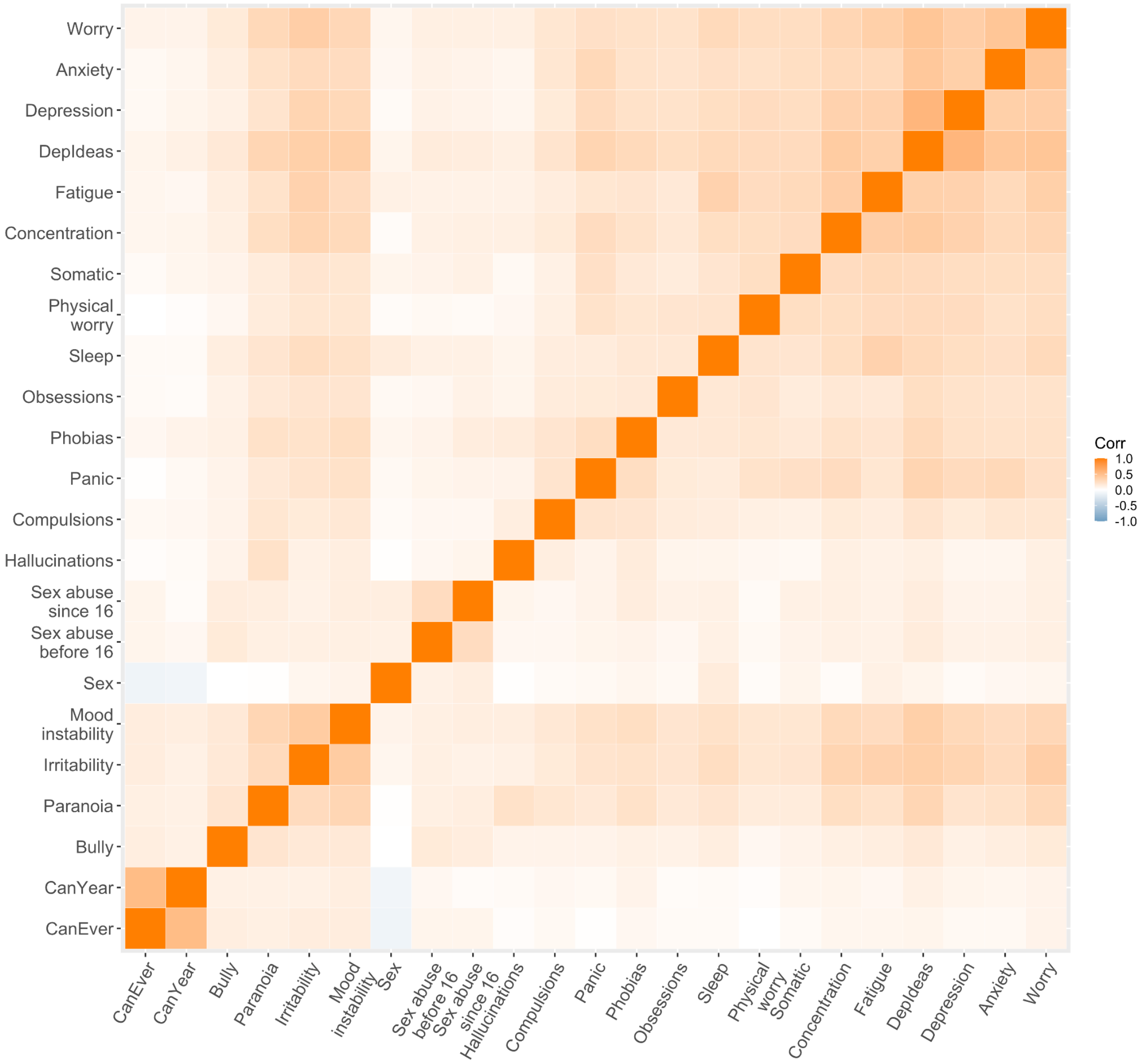

Supplementary Figure 1: Correlation plot of the full set of variables for 2007. Shades of orange correspond to positive associations, shades of blue to negative associations, and white represents the middle point of zero correlation.

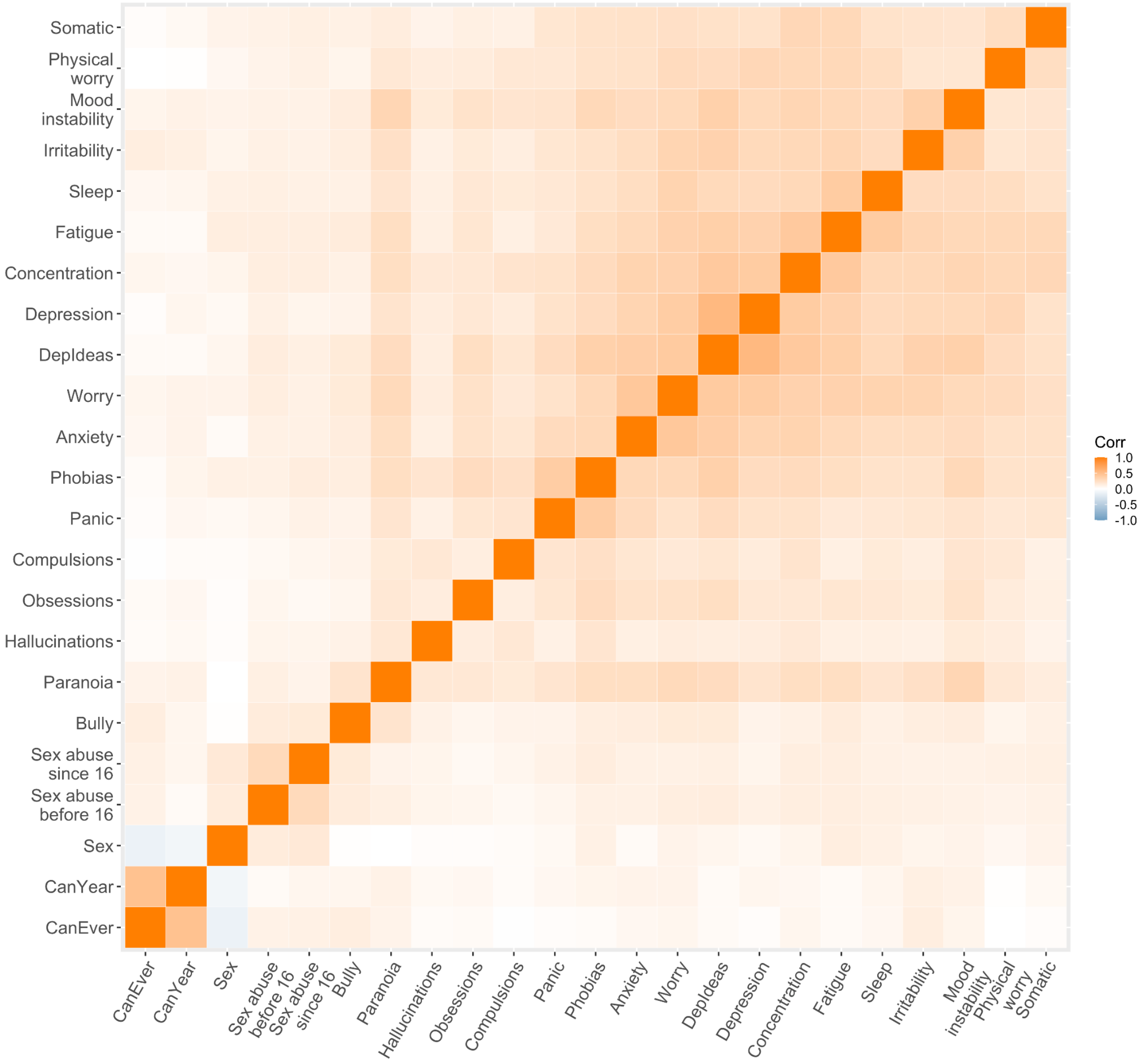

Supplementary Figure 2: Correlation plot of the full set of variables for 2014. Shades of orange correspond to positive associations, shades of blue to negative associations, and white represents the middle point of zero correlation.
